# 15017 Monoclonal antibody use in rheumatoid arthritis: an evaluation of medical expenditure

**DOI:** 10.1017/cts.2021.478

**Published:** 2021-03-30

**Authors:** Robert A. Kelly, Alice C. Ceacareanu, Zachary A.P. Wintrob

**Affiliations:** 1 Hartwick College; 2 ROAKETIN Inc.

## Abstract

ABSTRACT IMPACT: Younger patients receiving biologics for rheumatoid arthritis have higher medical expenditure. OBJECTIVES/GOALS: TNF inhibiting biologic disease modifying antirheumatic drugs are among the most highly regarded treatment options for rheumatoid arthritis (RA). We aimed at evaluating the medical and prescription costs associated with monoclonal antibody use vs. other RA treatment options in subjects diagnosed with RA. METHODS/STUDY POPULATION: Records from the Medical Panels Expenditure Survey (MEPS) database made available by the Agency for Healthcare Research and Quality were used to identify all RA subjects (n=_____). Demographics and MEPS-provided flags for RA were abstracted from the medical condition files for all the subjects surveyed (2008-2018). Prescribed biologics were identified based on generic and brand names following a manual review to detect any misspellings. Total medical expenses and prescription expenses were abstracted for all identified RA subjects. Subject were surveyed for two consecutive years, thus expenses were assessed for each of the two surveyed years. Costs were adjusted for inflation and expressed in 2018 dollars. The relationship between biologics use, cost and age or gender was evaluated by Fisher’s exact test. RESULTS/ANTICIPATED RESULTS: Most RA subjects did not use biologics. RA was more prevalent in women than in men with no significant correlation between sex and the use of biologics in year 1, year 2, or the combined years (p=.44, p=.63, and p=.65, respectively). Biologics users were found to be significantly younger (p<.001), with a mean of 52.8 years compared to 59 years in those who did not use biologics. The 95% confidence interval was 3.7 to 8.6 years younger than non-users. Total medical and prescription costs were higher for biologics users (p<.001) in all analyses. The mean prescription cost difference was $24,038 more per year for biologics users, and $26,296 more total medical expenses, CI $20,502-$27,230 and CI $21,947-$30,646, respectively. There was a trend for biologics users to have higher non-prescription medical expenses (p=.05). DISCUSSION/SIGNIFICANCE OF FINDINGS: Interestingly, biologics non-users had some extreme outliers with expenses far higher than any biologics users, possibly due to poorly controlled RA due to age and/or comorbidities. Yet, our most interesting findings are the higher use of biologics among younger RA subjects and the elevated costs of care being driven mainly by prescriptions cost.

